# The Association between Advanced Maternal Age and the Manifestations of Preeclampsia with Severe Features

**DOI:** 10.3390/jcm12206545

**Published:** 2023-10-16

**Authors:** Itamar Gilboa, Michael Kupferminc, Anat Schwartz, Yisca Landsberg Ashereh, Yariv Yogev, Avital Rappaport Skornik, Chagit Klieger, Liran Hiersch, Eli Rimon

**Affiliations:** 1 Tel Aviv Sourasky Medical Center, Tel Aviv 6423906, Israel; itamargi@tlvmc.gov (I.G.); kupferminc@tlvmc.gov.il (M.K.); anatsch3@gmail.com (A.S.); jiscaal@tlvmc.gov (Y.L.A.); yarivy@tlvmc.gov.il (Y.Y.); avitalsr@tlvmc.gov (A.R.S.); chagitk@tlvmc.gov (C.K.); liranh@tlvmc.gov.il (L.H.); 2 The Chaim Sheba Medical Center, Sackler Faculty of Medicine, Tel Aviv University, Tel Aviv 6997801, Israel

**Keywords:** advanced maternal age, preeclampsia with severe features, HELLP syndrome

## Abstract

This retrospective cohort study aimed to explore the association between advanced maternal age and the clinical manifestations as well as laboratory parameters of preeclampsia with severe features. This study included 452 patients who were diagnosed with preeclampsia with severe features. The clinical and laboratorial characteristics of patients with preeclampsia with severe features aged ≥40 years old (study group) were compared to those of patients aged <40 years old (control group). Multivariant analysis was applied to assess the association between advanced maternal age and the manifestations of preeclampsia with severe features, adjusting for the variables that exhibited significant differences between the study and control groups. The multivariate analysis revealed that a maternal age of ≥40 years old was an independent risk factor for acute kidney injury (OR = 2.5, CI = 1.2–4.9, *p* = 0.011) and for new-onset postpartum preeclampsia (OR = 2.4, CI = 1.0–5.6, *p* = 0.046). Conversely, a maternal age ≥ 40 years old was associated with a reduced risk of HELLP syndrome (OR = 0.4, CI = 0.2–0.9, *p* = 0.018) and thrombocytopenia (OR = 0.5, CI = 0.3–0.9, *p* = 0.016) compared to that of the patients < 40 years of age. In conclusion, this study demonstrates that maternal age is significantly associated with the clinical manifestations and laboratory parameters of preeclampsia with severe features, highlighting the importance of age-specific management.

## 1. Introduction

Over the last few decades, the increased use of assisted reproductive technology and socio-economic shifts changes have led to a rise in the rate of pregnant patients at an advanced maternal age (AMA), usually defined as either an age > 35 or an age > 40 years old [1,2,3]. Several studies have shown that AMA is a risk factor for various obstetric complications, such as gestational diabetes mellitus (GDM) [4,5,6], cesarean section (CS) [4,6,7], preterm delivery [5,6,8], and intrauterine growth retardation (IUGR) [4,5,6]. Notably, AMA is considered a major risk factor for the development of preeclampsia (PE) [3,4,6,8]. The incidence of PE with severe features, which complicates 0.5–1.6% of pregnancies [4,9], was also reported to be higher among patients with AMA compared to that of younger patients [4,8,9]. While several studies have confirmed the association between AMA and PE with severe features, the data regarding the association between AMA and unique characteristics of PE with severe features are quite limited.

We hypothesize that the clinical manifestations and laboratory parameters of PE with severe features may differ according to maternal age. We believe that this information will be relevant for the management of patients with PE and severe features. Thus, we aimed to investigate the association between AMA and the characteristics of PE with severe features by comparing the clinical manifestations and laboratory parameters of PE with severe features among patients ≥ 40 years old and patients < 40 years old.

## 2. Materials and Methods

### 2.1. Study Design and Participants

This was a retrospective cohort study of patients who gave birth after 24 0/7 weeks of gestation in a single, tertiary university-affiliated medical center in the years 2011–2019. We have conducted an extensive search within our database to identify the patients diagnosed with preeclampsia (PE) exhibiting severe features, and subsequently included those meeting the criteria in our analysis [10]. Patients were excluded if essential information concerning maternal demographics, obstetric parameters, PE characteristics, maternal and neonatal outcomes, and postpartum follow-up was absent. During the study period, the patients among whom PE with severe features occurred after 34 weeks of being pregnant were included. If PE with severe features occurred before 34 weeks, these patients were conservatively managed unless there were maternal or fetal indications for delivery [11]. All the data were collected from our institution’s computerized perinatal database.

### 2.2. Definitions

PE with severe features was defined as one of the following: systolic blood pressure ≥ 160 mm Hg or diastolic blood pressure ≥ 110 mm Hg taken on two occasions at least four hours apart, or elevated systolic blood pressure of 140–159 mm Hg or elevated diastolic blood pressure of 90–109 mm Hg taken on two occasions at least four hours apart. This included the presence of any of the following conditions: thrombocytopenia (platelet count ≤ 100,000/μL); elevated liver enzymes (alanine aminotransferase (ALT) or aspartate aminotransferase (AST) ≥ twice the upper level); severe persistent right-upper-quadrant/epigastric pain that is unresponsive to medication; an acute kidney injury (AKI) (defined as elevated serum creatinine > 1.1 mg/dL or doubling of serum creatinine in the absence of other renal disease); pulmonary edema or the new-onset of cerebral/visual disturbance (10). HELLP syndrome was defined via hemolysis (based on lactate dehydrogenase (LDH) > 600 IU/L, and/or serum bilirubin ≥ 1.2 mg/dL, and/or suggestive peripheral blood smear results), elevated liver enzymes (ALT and AST ≥ twice the upper level), and thrombocytopenia (platelet count ≤ 100,000/μL) [10]. New-onset postpartum PE was defined as PE which occurred within 6 weeks postpartum, excluding patients with antepartum PE and persistent PE [12]. Small for gestational age (SGA) was defined as infants with a birth weight below the 10th percentile for their gestational age using the local population’s live-born infant curves [13].

### 2.3. Outcome Measures

The primary objective of this study was to compare the clinical manifestations and laboratory parameters of PE with severe features among patients ≥ 40 years old (study group) and patients < 40 years old (control group). Therefore, we compared the prevalence of various clinical and laboratorial parameters, which constitute the criteria for PE with severe features in the study and control groups. Therefore, the clinical manifestations and laboratorial parameters of PE with severe features were defined as the depended variable and are summarized in the second table in Section 3. The data extracted from the database was gestational age (GA) at the onset of PE, maximal measured systolic and diastolic blood pressure, proteinuria, platelet count, the levels of ALT and AST, LDH and creatinine, clinical evidence for severe persistent right-upper-quadrant/epigastric pain, pulmonary edema or new-onset of cerebral/visual disturbance, and the occurrence of new-onset postpartum PE. The additional data collected was maternal age, gravidity, parity, body mass index (BMI) (calculated as weight (kg)/height (m^2^)), pre-gestational diabetes mellitus (DM), chronic hypertension, maternal smoking status, and mode of conception.

The secondary outcomes consisted of GA at delivery, mode of delivery, gestational diabetes mellitus (GDM), and the development of early onset PE prior to 34 weeks of gestation. The neonatal data included: gender, birth weight, one- and five-minutes Apgar scores, cord blood pH, amniotic fluid content, neonatal intensive care unit (NICU) admissions and length of NICU hospitalization, intraventricular hemorrhage (IVH), respiratory distress syndrome (RDS), apnea of immaturity, transient tachypnea of the newborn (TTN), hypoglycemia, jaundice, neonatal anemia, necrotizing enterocolitis (NEC), sepsis (positive blood or cerebrospinal fluid culture), and neonatal death within the first 28 days of life.

### 2.4. Statistical Analysis

The categorical variables were summarized as frequency and percentage. The normality of the continuous variables was tested using the Kolmogorov–Smirnov test, and variables were summarized as mean ± SD if normally distributed, or median and IQR if normality could not be assumed. Comparisons between the groups were performed with the Student *t*-test for continuous variables that are normally distributed and with the Mann–Whitney rank sum test for continuous variables that are not normally distributed. Chi-square and Fisher’s exact tests were used for comparing categorical variables. Multivariate logistic regression analysis was applied to investigate the association between maternal age and the characteristics of PE with severe features. The models included parameters with *p* < 0.05 in the univariate analysis including: IVF, smoking, and gestational DM. A *p*-value < 0.05 was considered statistically significant. Additionally, we conducted multivariate logistic regression analysis for HELLP and new-onset postpartum PE. All statistical analyses were performed using SPSS software (SPSS version 27, IBM, Chicago, IL, USA).

### 2.5. Ethical Approval

The study was conducted according to the guidelines of the Declaration of Helsinki and was approved by the local Institutional Review Board (Tel-Aviv Sourasky Medical Center IRB; protocol number 0284-0-TLV; date: July 2021).

## 3. Results

During the study period, there were 87,424 deliveries in our institution, of which 452 (0.5%) had PE with severe features. One hundred forty-five patients were ≥40 years old at the time of delivery (study group), and three hundred and seven patients were <40 years old (control group). The demographic and obstetric characteristics of the study and the control groups are presented in Table 1. The rates of IVF and smoking were higher in the study group compared to those of the control group.

The data on the various clinical and laboratory parameters comprising the criteria of severe features in the study and control groups are presented in Table 2. Epigastric pain was more common in the control group (28.7% vs. 19.3%, *p* = 0.034). The rates of headaches and visual changes were similar. HELLP syndrome was significantly more common in the control group (19.5% vs. 10.3%, *p* = 0.014). In contrast, patients ≥ 40 years old had a higher incidence of AKIs (20.7% vs. 10.4%, *p* = 0.003) compared to that of the patients < 40 years old.

Maternal morbidity and pregnancy complication data are presented in Table 3. Interestingly, the patients ≥ 40 years old had an increased rate of new-onset postpartum PE (12.4% vs. 6.8%, *p* = 0.049). The patients ≥ 40 years old also had higher rates of GDM (28.8% vs. 8.5%, *p* < 0.01) and of non-elective CS associated with a maternal indication (71.0% vs. 59.3%, *p* = 0.016) (Table 3).

The neonatal parameters were comparable in both groups (Table 4).

Multivariate logistic regression analysis (presented in Table 5) revealed that maternal age ≥ 40 years was an independent risk factor for an AKI (OR = 2.5, CI = 1.2–4.9, *p* = 0.011) and for new-onset postpartum PE (OR = 2.4, CI = 1.0–5.6, *p* = 0.046). Conversely, those with a maternal age ≥ 40 years old were found to have lower risk of HELLP syndrome (OR = 0.41, CI = 0.2–0.9, *p* = 0.018) and thrombocytopenia (OR = 0.5, CI = 0.3–0.9, *p* = 0.016) compared to that of the patients < 40 years of age.

## 4. Discussion

In the current study, we aimed to investigate whether PE with severe features in AMA patients, which is defined as a maternal age ≥ 40 years old, is associated with specific clinical manifestations and laboratory parameters. The incidence rate of PE with severe features in our population was 0.5%, which is similar to the incidence reported in previous studies [4,9]. Our main findings were: (1) patients with PE and severe features who are ≥ 40 years old are at an increased risk of developing an AKI and new-onset postpartum PE compared to that of the patients < 40 years old; (2) patients with maternal age ≥ 40 have a lower risk of developing HELLP syndrome and thrombocytopenia compared to that of the patients < 40 years old.

While several studies have documented an association between AMA and PE with severe features [4,8,9], there are limited data regarding the effect of maternal age on various characteristics of PE with severe features. Recently, Rymer-Haskel et al. [14], in a retrospective case–control study, compared the prevalence of various clinical and laboratory parameters among patients with PE > 35 years old and patients < 35 years old. In agreement with our results, the authors showed that patients with PE > 35 years old had a higher risk of developing an AKI (15.2% vs. 4.3%, respectively). Both the data reported by these authors and our findings are further supported by previous studies that have already reported an increase in the incidence of renal failure among AMA patients with or without PE [15,16]. It was suggested that older patients are more prone to developing renal insufficiency even if they still have normal pre-gestational kidney functions due to reduced renal reserves and the inability to adjust to physiological changes during pregnancy [17]. Our results are unique since the population of patients with PE with severe features was not specifically investigated in these studies. Obviously, once PE with severe features occurs among AMA patients, the risk of developing an AKI further increases, as endothelial injuries, which affect renal function dramatically, are more prominent [18,19]. Therefore, it is not surprising that we found even higher rates of AKIs in both the study and control groups compared to those in previous studies [14,15].

We have also shown that maternal age ≥ 40 years old is an independent risk factor for new-onset postpartum PE, which explains the higher rates of hospital readmissions in this group of patients. Our findings are in concordance with prior observations reporting an association between postpartum PE and AMA [20,21,22]. Interestingly, Sibai et al. [21] and Vilchez et al. [22] have suggested that the pathophysiology of postpartum PE is related to the shift of large amounts of fluids from the extravascular space to the intravascular space, which, in turn, leads to volume overload-induced hypertension. Importantly, this phenomenon is more prominent among patients with PE with severe features due to underlying endothelial damage and the increased amounts of fluids that accumulate during pregnancy in the extravascular space [21,22]. Taken together, our findings regarding the increased rates of new-onset postpartum PE among patients in the study group are logical given their risk factors for postpartum PE. Therefore, patients ≥ 40 years old should be under careful surveillance during the postpartum period.

In the current study, the patients under 40 years old who developed PE with severe features had a higher risk of developing HELLP syndrome compared to that of the patients ≥ 40 years old. This finding was also strengthened by additional findings, including significantly lower levels of platelets, increased rates of elevated AST and ALT levels, and an increased rate of epigastric pain in patients under 40 years old. In contrast, previous studies have linked AMA with an increased risk of HELLP syndrome [23,24]. However, this association was documented in studies that investigated the relationship between the incidence of HELLP syndrome and maternal age in the general population. Therefore, a possible explanation for the inconsistencies is that our study focused on a highly specific population of patients who have already experienced PE with severe features. We could not find a study in the literature that specifically investigated the risk of patients with preeclampsia with severe features of developing HELLP syndrome, and hence, our findings are unique. Moreover, in line with our results, a recent study found that patients with PE < 35 years old had higher rates of elevated liver enzymes, low platelet levels, and epigastric pain compared to those of AMA patients, but these differences did not reach statistical significance, probably because the study group included patients with mild PE [14].

The neonatal parameters were comparable in both groups, including comparable rates of birth weight below the tenth percentile and birth weight below the third percentile. Previous studies have shown that AMA is associated with an increased risk of SGA [4,6,25,26]. However, it should be noted that our study focused on a highly selective group of patients with PE with severe features who already have an increased risk of IUGR compared to that of AMA patients without PE [27]. Indeed, in our study, the rates of birth weight below the tenth percentile and birth weight below the third percentiles in both groups were already higher than the values reported for AMA patients without PE [4,6,7,25,26], which is probably due to a severe underlying placental injury in the patients with PE with severe features [28]. Therefore, we suggest that in the presence of a severe placental injury in this highly selected population of patients with PE with severe features, AMA might not have any additional effect on the rate of IUGR.

Our study has several notable strengths. The main strength of our study was the relatively large cohort of the highly selected population of patients with PE with severe features who gave birth in a single tertiary medical center. Although the study included women who gave birth in our institution during the years 2011–2019, we defined PE with severe features according to the new ACOG guidelines [13] to keep our recommendations updated.

On the other hand, a major limitation of this study is its’ retrospective design and limited data on additional prognostic markers, which could have been beneficial in drawing a more accurate model. Another limitation of the study is the lack of information on various prognostic parameters, such as gestational age at the diagnosis of gestational hypertension, familiarity with PE, history of PE, angiogenic markers, uric acid level, and serum-uric-acid-to-serum-creatinine ratio. Additionally, the absence of randomization and control over the variables poses challenges in accounting for potential confounders, which might have influenced the observed associations.

## 5. Conclusions

The aim of this study was to investigate the association between maternal age and the clinical manifestations and laboratory parameters of PE with severe features. The findings in the current study add to the literature by presenting a possible association between maternal age and specific manifestations of PE with severe features. We presented unique findings that suggest that the presentations of PE with severe features are different depending on maternal age ≥ 40 years old or <40 years old. These findings suggest that clinicians should take into consideration maternal age as a potential risk factor for specific complications and adjust the follow-up protocol according to maternal age. Specifically, more caution should be used in the management of patients with PE with severe features > 40 years old regarding AKIs and new-onset postpartum PE. As for the patients < 40 years old, clinicians should be aware that they have a higher risk of developing HELLP and its associated complications. Further studies are required to support these findings.

## Figures and Tables

**Table 1:** Maternal demographics and obstetrical characteristics in the study and control groups.

	Patients’ Age <40 Years	Patients’ Age ≥40 Years	*p*-Value
No. of patients	307	145	
Age at time of delivery (years)	31.7 ± 3.2	44.4 ± 3.4	<0.001
Nulliparity	201 (65.5)	94 (64.8)	0.893
Multiple gestation	41 (13.4)	21 (14.5)	0.745
IVF mode of conception	37 (12.1)	103 (71.0)	<0.001
Pregestational BMI (kg/m^2^) ≥ 30	25 (9.3)	20 (15.6)	0.063
Gestational weight gain (kg)	12.5 ± 6.0	11.6 ± 8.6	0.947
Chronic disease			
Chronic hypertension	27 (8.8)	19 (13.1)	0.161
Pregestational diabetes	9 (2.9)	3 (2.1)	0.594
Systemic lupus erythematous	2 (0.7)	1 (0.7)	0.963
Anti-phospholipid syndrome	5 (1.6)	4 (2.8)	0.422
Hereditary hypercoagulation	3 (1.0)	4 (2.8)	0.152
Smoking	5 (1.6)	7 (4.8)	0.048
Corticosteroid administration	92 (30.0)	52 (35.9)	0.209
Gestational age at delivery (weeks)	35.5 ± 3.5	35.7 ± 3.1	0.601
Delivery < 34 weeks	87 (28.3)	38 (26.2)	0.637
Delivery < 37 weeks	188 (61.2)	86 (59.3)	0.695

Data are presented as n (%) or mean ± SD; IVF, in vitro fertilization; BMI, basic metabolic index.

**Table 2:** Prevalence of parameters defining PE with severe features in the study and control groups.

	Patients’ Age <40 Years	Patients’ Age ≥40 Years	*p*-Value
No. of patients	307	145	
Elevated blood pressure	217 (70.7%)	110 (75.9%)	0.251
Systolic blood pressure (mm\Hg)	167.6 ± 20.9	170.5 ± 21.1	0.072
Diastolic blood pressure (mm\Hg)	96.9 ± 12.9	94.5 ± 13.1	0.026
Mean arterial pressure (mm\Hg)	120.5 ± 13.8	119.8 ± 14.0	0.584
Symptoms	162 (52.8%)	64 (44.1%)	0.087
Headache	73 (23.8%)	40 (27.6%)	0.383
Epigastric pain	88 (28.7%)	28 (19.3%)	0.034
Visual changes	40 (13.0%)	22 (15.2%)	0.536
Thrombocytopenia	107 (34.9%)	30 (20.7%)	0.002
Platelets (thousands/µL)	127.7 ± 58.6	146.3 ± 55.0	<0.001
Elevated liver enzymes (≥twice upper level)	132 (43.0%)	50 (34.5%)	0.085
ALT (IU/L)	51 (21–124)	32 (18–94)	0.040
AST (IU/L)	61 (32–131)	42 (28–107)	0.025
Acute kidney injury	32 (10.4%)	30 (20.7%)	0.003
Pulmonary edema	10 (3.3%)	5 (3.4%)	0.916
HELLP	60 (19.5)	15 (10.3)	0.014
Eclampsia	7 (2.3)	2 (1.4)	0.522

Data are presented as n (%), mean ± SD or median IQR (25–75%); HELLP, hemolysis, elevated liver enzymes and low platelets; MAP, mean arterial pressure; ALT, alanine aminotransferase; AST, aspartate aminotransferase.

**Table 3:** Maternal morbidity and pregnancy complications in the study and control groups.

	Patients’ Age <40 Years	Patients’ Age ≥40 Years	*p*-Value
No. of patients	307	145	
Early PE < 34 weeks	102 (33.2%)	55 (37.9%)	0.327
Placental abruption	6 (2.0%)	3 (2.1%)	0.935
New-onset postpartum PE	21 (6.8%)	18 (12.4%)	0.049
Gestational diabetes mellitus	26 (8.5%)	33 (28.8%)	<0.001
Cesarean section	238 (77.5%)	125 (86.2%)	0.030
Non-elective CS	226 (73.6%)	118 (81.4%)	0.071
Neonatal indication	44 (14.3%)	15 (10.3%)	0.240
Maternal indication	182 (59.3%)	103 (71.0%)	0.016
Elective CS	14 (5.0%)	7 (5.9%)	0.706
Length of hospitalization (days)	5.1 ± 1.9	5.9 ± 2.7	<0.001
Repeated hospitalization	7 (2.3%)	10 (6.9%)	0.016
red blood cells transfusion ≥ 1	28 (9.1%)	9 (6.2%)	0.292
red blood cells transfusion ≥ 4	4 (1.3%)	0 (0%)	0.162
ICU admission	19 (6.9%)	5 (3.4%)	0.222

Data are presented as n (%) or mean ± SD; PE, preeclampsia; CS, cesarean section; ICU, intensive care unit.

**Table 4:** Neonatal outcomes in the study and control groups.

	Patients’ Age <40 Years	Patients’ Age ≥40 Years	*p*-Value
No. of patients	347	167	
Gender—male	148 (42.7%)	76 (45.4%)	0.541
Birthweight (gr)	2140 (1632–2680)	2245 (1745–2767)	0.148
Birthweight percentile	30.9 ± 26.7	36.7 ± 26.8	0.021
Birthweight < 10%	103 (33.6%)	36 (24.8%)	0.061
Birthweight < 3%	27 (7.8%)	17 (10.17%)	0.382
Amniotic fluid-Meconium	26 (7.5%)	6 (3.6%)	0.085
5 min Apgar score < 7	24 (6.9%)	16 (9.6%)	0.291
Umbilical cord pH < 7.1	6 (1.7%)	5 (3.0%)	0.356
NICU	53 (15.3%)	29 (17.4%)	0.544
NICU length of hospitalization (days)	5.8 (17.1%)	5.2 (13.5%)	0.556
RDS	45 (13.0%)	25 (15.0%)	0.535
Apnea	28 (8.1%)	10 (6.0%)	0.228
TTN	11 (3.2%)	5 (3.0%)	0.914
Hypoglycemia	39 (11.2%)	24 (14.4%)	0.311
Jaundice	58 (16.7%)	35 (21.0%)	0.242
Necrotizing enterocolitis	19 (5.5%)	6 (3.6%)	0.353
Intraventricular hemorrhage	10 (2.9%)	9 (5.4%)	0.158
Sepsis	2 (0.6%)	0 (0%)	0.326
Neonatal anemia	43 (12.4%)	20 (12.0%)	0.893
Perinatal death	2 (0.6%)	3 (1.8%)	0.187

Data are presented as n (%), mean ± SD or median IQR (25–75%). SGA, small for gestational age; NICU, neonatal intensive care unit; RDS, respiratory distress syndrome; TTN, transient tachypnea of a newborn.

**Table 5:** A multivariate analysis of the association between maternal age ≥ 40 years and manifestations of PE with severe features.

	Adjusted Regression Age ≥ 40 Years			Unadjusted Regression Age ≥ 40 Years		
	*p*-Value	CI	aOR (95% CI)	*p*-Value	CI	uaOR (95% CI)
HELLP	0.5	0.3–0.9	0.016	0.4	0.2–0.9	0.018
Thrombocytopenia	0.5	0.3–0.8	0.002	0.5	0.3–0.9	0.016
Elevated liver enzymes	0.7	0.5–1.1	0.086	0.7	0.4–1.2	0.182
AKI	2.2	1.3–3.9	0.004	2.5	1.2–4.9	0.011
New-onset postpartum PE	1.9	0.9–3.7	0.052	2.4	1.0–5.6	0.046

Adjusted for IVF, smoking, and GDM.

## Data Availability

Data is unavailable due to privacy or ethical restrictions.
